# Oxidative stress vulnerability and cellular conditioning in neural precursors from schizophrenia patients

**DOI:** 10.1192/j.eurpsy.2026.10735

**Published:** 2026-07-31

**Authors:** C. Cachán-Vega, R. Villa Díez, E. A. Povedano Suárez, J. J. Martínez Jambrina, A. Coto-Montes, Y. Potes

**Affiliations:** 1 Faculty of Medicine, Morphology and Cell Biology, University of Oviedo; 2 Instituto de Investigación Sanitaria del Principado de Asturias (ISPA); 3 Instituto de Neurociencias del Principado de Asturias (INEUROPA), Oviedo; 4 Hospital San Agustín, Avilés, Spain

## Abstract

**Introduction:**

Cellular responses to oxidative stress have been consistently implicated in the pathophysiology of schizophrenia (SCH), potentially impacting key cellular processes. Cellular proliferation and metabolic viability, which are directly linked to redox homeostasis, remain poorly understood in this context despite their recognized importance for neuronal health.

**Objectives:**

The aim of this study was to investigate whether neural precursors from SCH patients display altered proliferative capacity and cell viability under oxidative stress, to identify disease-related differences in stress responses that could serve as potential therapeutic targets.

**Methods:**

Neural precursors were isolated from olfactory neuroepithelium biopsies obtained from SCH patients (N=6) and matched healthy controls (N=5) enrolled in the MINDS cohort (Mental Illness Neuroepithelium Derived Samples). Cells were exposed to hydrogen peroxide (H₂O₂) as an inducer of oxidative stress, and cellular proliferation and viability were assessed using the SRB and MTT assays at 0 h, 24 h, 48 h, and 72 h post-treatment.

**Results:**

Neural precursors from SCH patients displayed a slight reduction in proliferation compared to controls starting at 48 h. When exposed to an oxidative stress environment induced by H₂O₂, both SCH and control cells showed an approximate 50% decrease in proliferation relative to untreated conditions, with differences between groups becoming evident at 24 h post-exposure, when SCH-derived cells exhibited a more pronounced reduction in proliferative capacity. In parallel, cell viability remained stable over time under basal conditions in both groups, but following H₂O₂ exposure a marked decline was observed at 24 h in both SCH and control cells. Interestingly, while viability continued to decrease in controls, SCH-derived cells displayed a progressive recovery over time, ultimately showing higher viability compared to controls

**Conclusions:**

Exposure to an oxidative stress environment impairs the proliferative potential of SCH-derived neural precursors, while simultaneously revealing an adaptive response characterized by enhanced survival capacity. This may reflect a cellular “conditioning” effect, where chronic stress promotes increased resistance to oxidative damage, as described in other contexts such as chronic training (PMID: 22215375) and aging (PMID: 38459002).

**Disclosure of Interest:**

None Declared

